# High-fidelity discrete modeling of the HPA axis: a study of regulatory plasticity in biology

**DOI:** 10.1186/s12918-018-0599-1

**Published:** 2018-07-17

**Authors:** Hooman Sedghamiz, Matthew Morris, Travis J. A. Craddock, Darrell Whitley, Gordon Broderick

**Affiliations:** 1 Center for Clinical Systems Biology, Rochester General Hospital, 1425 Portland Ave, Rochester, 14621 US; 2 Biomedical Engineering Department, Rochester Institute of Technology, One Lomb Memorial Drive, Rochester, 14623 US; 3 Institute for Neuro Immune Medicine, Nova Southeastern University, 8501 SW 124th Avenue, Davie, 33183 US; 4 Departments of Psychology and Neuroscience, Computer Science, and Clinical Immunology, Nova Southeastern University, 8501 SW 124th Avenue, Davie, 33183 US; 5 School of Computer Science, Colorado State University, University Ave, Fort Collins, 80521 US

**Keywords:** Logical Modeling, Multi-valued discrete simulation, biological regulatory feedback, HPA axis, Regulatory stability, HPA axis plasticity

## Abstract

**Background:**

The hypothalamic-pituitary-adrenal (HPA) axis is a central regulator of stress response and its dysfunction has been associated with a broad range of complex illnesses including Gulf War Illness (GWI) and Chronic Fatigue Syndrome (CFS). Though classical mathematical approaches have been used to model HPA function in isolation, its broad regulatory interactions with immune and central nervous function are such that the biological fidelity of simulations is undermined by the limited availability of reliable parameter estimates.

**Method:**

Here we introduce and apply a generalized discrete formalism to recover multiple stable regulatory programs of the HPA axis using little more than connectivity between physiological components. This simple discrete model captures cyclic attractors such as the circadian rhythm by applying generic constraints to a minimal parameter set; this is distinct from Ordinary Differential Equation (ODE) models, which require broad and precise parameter sets. Parameter tuning is accomplished by decomposition of the overall regulatory network into isolated sub-networks that support cyclic attractors. Network behavior is simulated using a novel asynchronous updating scheme that enforces priority with memory within and between physiological compartments.

**Results:**

Consistent with much more complex conventional models of the HPA axis, this parsimonious framework supports two cyclic attractors, governed by higher and lower levels of cortisol respectively. Importantly, results suggest that stress may remodel the stability landscape of this system, favoring migration from one stable circadian cycle to the other. Access to each regime is dependent on HPA axis tone, captured here by the tunable parameters of the multi-valued logic. Likewise, an idealized glucocorticoid receptor blocker alters the regulatory topology such that maintenance of persistently low cortisol levels is rendered unstable, favoring a return to normal circadian oscillation in both cortisol and glucocorticoid receptor expression.

**Conclusion:**

These results emphasize the significance of regulatory connectivity alone and how regulatory plasticity may be explored using simple discrete logic and minimal data compared to conventional methods.

**Electronic supplementary material:**

The online version of this article (10.1186/s12918-018-0599-1) contains supplementary material, which is available to authorized users.

## Background

The Hypothalamic-Pituitary-Adrenal (HPA) axis is one of the most fundamental components of the body in regulating the response to stress. Due to its important regulatory role, it is no surprise that the HPA axis has been associated with a number of complex chronic diseases such as Gulf War Illness (GWI) and Chronic Fatigue Syndrome [1–3]. Initially, stress is perceived by the central nervous system (CSN) and a pulse is transmitted to the hypothalamus to release corticotropin-releasing hormone (CRH) into the pituitary gland (PA) in the mid-brain. The pituitary initiates release of adrenocorticotropin hormone (ACTH) into the blood stream where it signals to the adrenal cortex to respond in turn and release cortisol (CORT) into the blood stream. CORT has broad effects across the body where it binds to glucocorticoid receptors (R) and in a negative feedback suppresses ACTH secretion [4]. Previously, models of the HPA axis have been formulated as sets of Ordinary Differential Equations (ODE) [5, 6], delay differential equations (DDEs) [7, 8] or as discrete Boolean (BN) networks [9]. Early work by our group extended the BN formalism to a fixed 3-state logic to provide additional detail [10]. While BN models are able to partially generate cyclic attractors [11], in the case of HPA axis, the complexity in the behavior of CORT (e.g. multi-level range) cannot be modeled in either the BN or extended BN framework. Therefore, in this study, we employ a fully generalized discrete network formalism introduced by Thomas [12] and use different updating schemes such as synchronous, asynchronous and priority updating with memory in order to recover bi-stable attractors in HPA axis behavior with minimal parameter selection. Our contribution is twofold; a model tuning algorithm that ensures alignment of model behavior with an expected qualitative outcome (e.g. cyclic attractor) and a new updating scheme based on assignment to a priority class with a memory of previous states that accounts for historical actions of the model. Our proposed tuning algorithm selects a set of logical parameter values that guarantee a target behavior such as the presence of limit cycles. This is done based on the identification of isolated positive and negative feedback loops in the regulatory signaling networks.

To illustrate the properties of this Generalized Discrete Formalism we construct a basic model of the HPA axis and compare the predictions against behaviors obtained using a much more detailed set of conventional ODEs. We show that the multi-level discrete logic proposed in this work accurately reproduces the bi-stable oscillatory behavior predicted by Kim et al. [7]. Moreover, we show that externally applied stress can temporarily collapse the attractor space to specific states or sets of states that may serve to re-initiate the system under an alternate homeostatic program after stress has dissipated. The range of these intermediate stress-potentiated states and their location are dependent upon the set of logic parameters imparted by HPA axis tone. We propose that knowledge of these “gateway” states may inform on potential mechanisms of onset for many of these stress-mediated illnesses, an aspect which remains poorly understood. Having a control subject under challenge access a stress-enabled state that is normally observed in chronic HPA axis dysfunction would suggest that such states act as a stepping stone in the sequence of onset events leading to persistent illness. Similarly, we show that an externally applied therapeutic agent, in this case a glucocorticoid receptor blocker, may render a given persistent regulatory program unstable and favor return to the original homeostatic regime. This plasticity of the attractor space suggests that systems such as these continuously adapt the repertoire of regulatory programs available to ensure stable behavior as an adaptive response to changes in environmental cues.

## Results

In this section, we illustrate how the bi-stability in HPA behavior, predicted by a much more elaborate ODE model proposed by Kim et al. [7] in the absence of external perturbations, may be recovered using this compact discrete formalism under various updating schemes. Secondly, we simulate how an external stressor may facilitate the migration from one stable regulatory cycle to another potentially pathogenic regulatory mode. Then, we assess the robustness of the attractors by introducing stochasticity in the state transition functions. Finally, we demonstrate how an external intervention, such as a R antagonist, might be applied to promote recovery of a more desirable circadian rhythm by rendering the alternate cycle dynamically unstable.

### Parameter identification

As described in greater detail in the “Methods” section, the current discrete model uses parameters defining the relative contextual weight of stimulatory and inhibitory signals (*K* values) and the threshold of activation *θ* required for a response to be produced. The HPA axis is one of the better studied physiological regulatory axes and its oscillatory [3, 7] and bi-stable [5–7] dynamic behavior has been well documented and these attributes served here as constraints for the identification of parameter values. Specifically, it has been shown that multi-stability [13, 14] and cyclical behavior require positive and negative feedback loops respectively. Therefore, in order to guarantee that the HPA model supports bi-stable cyclic attractors, its topology must contain at least one negative and one positive feedback loop. In addition, the feedback loops must be functional. Functional status is determined by assignment of logical values (K). Intuitively, our method first analyzes the topology of the network to identify feedback loops and their corresponding parity [15], and then exhaustively checks whether different values of K would make such feedback loops functional (see Additional file 1 for more details). Out of 65536 logical combinations of *K* values available to this model configuration, we found that only one parameterization (see Table 1) was able to reproduce bi-stable cyclic attractors. The values of activation threshold theta were necessarily 1 for single output elements but in the case of dual output nodes Cort and R each output received a threshold value that would ensure that their corresponding feedback loops became active. This parameter identification was performed only with respect to those parameters defining the behavior of CRH, ACTH, CORT and R. The perceived severity of an environmental threat is highly subjective, varying greatly from person to person based on a range of factors including genetic predisposition and life experiences. One might expect some individuals, for example combat veterans, being hyper-aware and responding more readily and more intensely to a stressor. In an attempt to capture and accommodate some of this variability in the perception of environmental stress we performed a separate model calibration for this model input after first finding a parameterization of the internal components (e.g. CRH, ACTH, CORT, R)of the model at rest in the absence of an external stimuli (Additional file 2).

**Table 1:** Feasible logical parameters (K) generating bi-stable cyclic attractors along with their equivalent logical equations (see [63] for more details on how these equations might be further simplified)

Component	Logical values(Kinetic Ratios)	Logical Equivalence
CRH (*i*=2)	*K*_2*∅*_=0, *K*_24_=1	*CRH*::(*CORT*⇔0).
ACTH (*i*=3)	*K*_3*∅*_=0, *K*_32_=1, *K*_35_=1, *K*_3.25_=1	*ACTH*::(*CRH* *AND* ¬(*R*⇔0)) *OR* (¬*CRH* *ANDR*⇔0) *OR*(*CRH* *ANDR*⇔0).
CORT (*i*=4)	*K*_4*∅*_=0, *K*_43_=2	*CORT*⇔2::*ACTH*.
R (*i*=5)	*K*_5*∅*_=0, *K*_54_=1, *K*_55_=2, *K*_5.45_=2	*R*⇔2::(*R*⇔2)*OR*(*R*⇔2 *AND* *CORT*⇔2).*R*⇔1::*CORT*⇔2*AND* ¬(*R*⇔2).

Note that Stress is not mentioned in this table since it has no input interaction. The binary entities (e.g. CRH and ACTH) are denoted by conventional logical notations (e.g. ¬*ACTH*::*ACTH*⇔0)

### HPA axis behavior in isolation

**Fig. 1:**
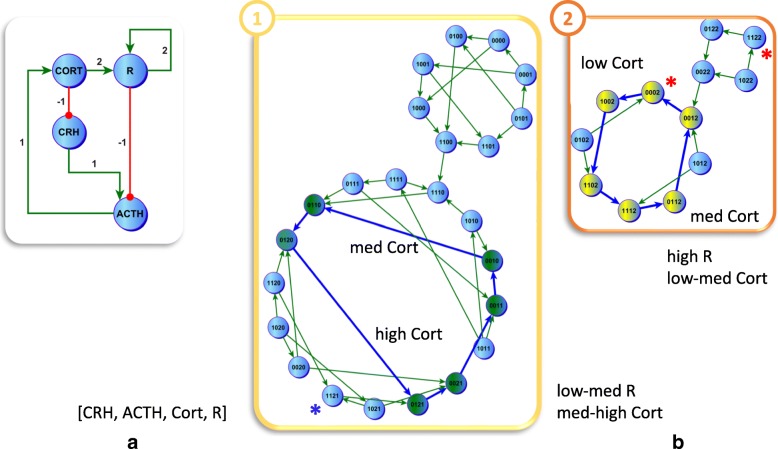
Recovering Multiple Regulatory Cycles for the HPA Axis. Applying a Priority Class updating scheme to a multi-level regulatory logic model of the hypothalamic-pituitary- adrenal (HPA) axis (**a**) produced a state transition graph (STG) in [CRH, ACTH, Cort, R] supporting two stable oscillatory cycles (**b**). The regulatory mode labeled as Healthy (green state nodes) supports oscillation in the state of glucocorticoid receptor R from low to medium expression with cortisol expression (Cort) oscillating from mid to high expression levels. Conversely, in the second regulatory regime Cort expression levels oscillate at the lower end of the range with R persistently over-expressed (yellow nodes). Note that in absence of external disturbances (assumed here), once the system has settled in one or the other of these cycles it will remain locked in that regulatory mode since there are no state transition edges supported by the circuitry that connect these separate attractors or their basins. States identified with an asterisk correspond to stationary states to which the system collapses under a chronic external stress (Figurer 3) that overlap with the oscillatory regime where cortisol spans the lower range (red) or the higher range of expression (blue)

A discrete generalized version of the HPA axis is illustrated in Fig. 1a. Note that the dimerized R (RD) and native R used by Gupta et al. [5] are modeled here as a single node R resulting in only 4 state variables in this variant of the model. The edges are labeled with the threshold of the interaction *θ*_*ij*_ at which they become active. In a first analysis we apply asynchronous simulations where a state node in the State Transition Graph (STG), describing the sequence of system states as they evolve across time, might have more than one successor. These support two complex singular or cyclic attractors (Additional file 3) in state variables [*CRH,ACTH,CORT,R*] respectively. While CORT levels oscillate across the full range of expression in both cycles, these regulatory modes differ significantly with respect to the expression of R which in one case remains overexpressed or saturated (upper limit 2). Similarly, synchronous updating of the state variables (Additional file 4) supports 4 cyclic attractors where CORT again oscillates between its maximum and minimum expression values. In two of these attractors we again notice the persistent overexpression of R.

Finally, we applied a priority class updating with memory where we separated the state variables into two classes namely fast and slow state transition or updating, based on their relative kinetics. Because of their anatomical proximity in the mid-brain, we placed CRH, ACTH and R in the fast group giving these state variables a first priority of update (Table 2). As the adrenal gland resides in the periphery and, CORT is released into the general circulatory compartment, this state variable was assigned to the slow group. Results of this stratification are shown in Fig. 1b (simplified in Additional file 5). As with fully asynchronous updating, we recover two singular or cyclic attractors. However, in this case CORT oscillates in a split range. In one cyclic attractor CORT oscillates at the high end of its range (1−2), with expression of R also oscillating, while in the second CORT oscillates at the low end of the range (0−1) of expression with R being saturated or persistently overexpressed.

**Table 2:** Frequency of Update (*d*_*i*_) for the priority with memory update

Component	*d*_*i*_
CRH (*i*=2)	*d*_2_=1
ACTH (*i*=3)	*d*_3_=1
CORT (*i*=4)	*d*_4_=4
R (*i*=5)	*d*_5_=1

Note that Stress is not mentioned in this table since it has no input interaction

### Simulating environmental challenge

**Fig. 2:**
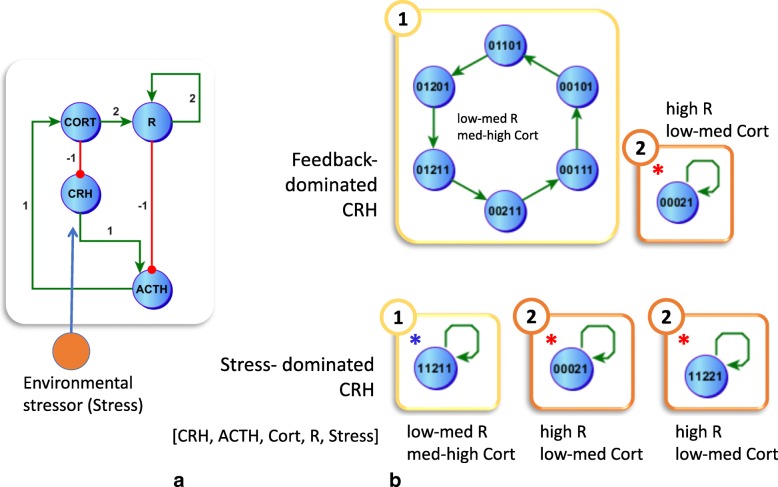
A Stress-mediated Collapse of Regulatory Repertoire. Introducing an environmental stressor (orange node) into the HPA regulatory circuit (**a**) alters the stable attractor space in [CRH, ACTH, Cort, R, Stress] such that cycles supported at rest collapse (**b**). Under normal regulatory feedback tone and sensitivity, stress serves to galvanize the Healthy regulatory cycle while also allowing for transition to a pathologic stationary point (red asterisk). Alternatively, in the case of a heightened sensitivity to stress the previous stability landscape collapses altogether to 3 stationary points, one belonging to the medium to high range cortisol cycle (blue asterisk) and two belonging to the low to medium range cortisol regime (red asterisk)

In an ideal protective environment, without any external disturbances such as stress, the two stable attractors recovered under a compartmentalized asynchronous updating in the previous section have no overlap, that is they do not share any common states transient or otherwise. As a result, it is impossible to migrate from one stable regime to the other (Fig. 1b). Of course this idealized sensory deprivation is not representative of the everyday world and we hypothesize that adding environmental factors to the model circuit may alter the regulatory landscape in a way that might facilitate migration from one attractor to the other. To test this hypothesis, we introduce a generic stressor that directly stimulates CRH synthesis and release (Fig. 2a) as a new environmental input or exogenous state variable to the model. Our results indicate that activation of this external stressor remodels the stability landscape in a way that may support the migration from one attractor to another. Applying stress directly to CRH, while keeping all other model parameters constant (unperturbed model) resulted in the identification of 16 parameter sets (Additional file 2) describing stress-CRH interactions supporting coherent dynamic behavior. Of these solutions 4 parameter sets supported 2 cyclic attractors, 4 sets supported 1 cyclic attractor and a stationary point, and the remaining 8 solutions supported only stationary points, that is those attractors containing only a single state. Further examination showed that among these, only 4 parameter sets supported biologically plausible behavior for CRH when stress was absent and only 2 supported expected HPA axis behavior when stress is present. These 2 final parameter sets supported stable states existing under persistent stress (Fig. 2b) that straddled the stable states available in the absence of stress, or with the HPA axis at rest (Fig. 1b). Specifically, the stationary point [0002] and [1122] both overlap with the cyclic attractor in the unstimulated system where R is chronically over expressed (Fig. 1b) trapping the system in that cycle when stress is removed.

### Inducing regulatory recovery

**Fig. 3:**
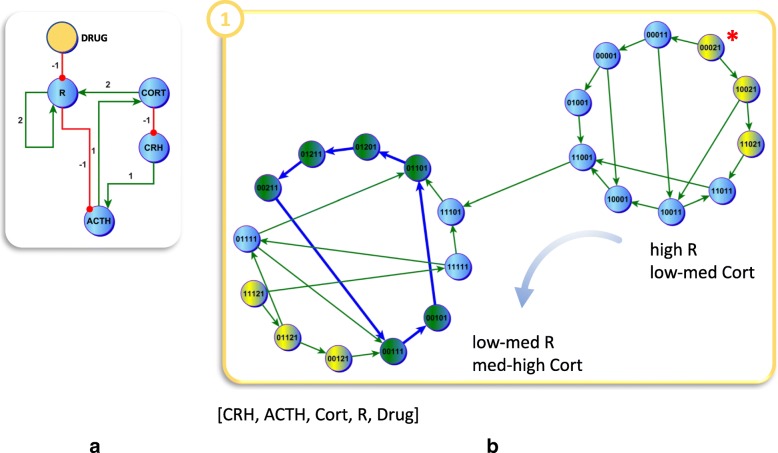
Shifting Stability in Favor of a Target Regulatory Program. As in Fig. 2, the introduction of an external factor, in this case a therapeutic agent (Drug; yellow node) inhibiting the expression of R (**a**), again alters the stability landscape significantly such that the previously stable pathological (low to medium cortisol expression) oscillatory regime, and its component stationary points supported under conditions of persistent stress (red asterisk), is now dynamically unstable (**b**). Indeed, under a therapeutic blockade of R the only stable regime in [CRH, ACTH, Cort, R, Drug] consists of the healthy (medium to high cortisol expression) attractor (including the subset of overlapping component states that remained available under untreated conditions of chronic stress, blue asterisks)

Consistent with the nature of these attractors, removal of the triggering insult does not reverse the condition. Interrupting this dynamically stable cycle of chronically under-expressed CORT will require another external perturbation. Here we simulate a reverse scenario where the corresponding over-expression of R is inhibited by an externally applied pharmaceutical antagonist (Fig. 3a). While an exhaustive evaluation of rescue strategies based on single-target interventions suggested that inhibition of CRH would also succeed, the latter is less widely used and somewhat more novel [16]. For this reason we focused this proof-of-principle on the more common inhibition of glucocortiocoid receptor R [17]. Initiating the simulations from any state in the chronic hypocortisolic attractor, we simulated the trajectory that the system might follow in order to migrate back to the target healthy state (Fig. 3b). Applying this idealized inhibitor of R we found that the attractor landscape changed such that the chronic low-level oscillatory regime for CORT facilitated by the persistent overexpression of R became dynamically unstable. Indeed, under these conditions the only stable regime remaining involved oscillation of R and CORT within desired ranges.

### Robustness of attractors with respect to stochasticity

In the previous sections, we show that this regulatory model of the HPA axis can in principle accommodate 2 stable oscillatory attractors and that under the influence of different environmental factors it might be possible to migrate from one regime to the other. While we confirm that it is possible to escape a given attractor it is also of interest to know how difficult this escape might be or in other words how strong an attraction is exerted by a given regulatory regime. We explore this by conducting 1000 repeated Monte Carlo simulations for each of the 36 possible initial states supported by the network, where we applied random errors *ε*=0.05 to the HPA regulatory logic in the following three numerical experiments:

- *Isolated wild type*: In this case, we performed repeated wild-type simulations of the network in isolation (that is without any external perturbation by environmental factors). Simulations of the HPA network were initiated at random states around both attractors. Statistics describing the resulting frequency of occurrence of the final resting states were used to reconstruct the transition matrix for each of the attractors.
- *Chronic environmental stress*: In this case, we simulated a chronic stress scenario in order to see how the transition probabilities separating attractors change in this new landscape. Specifically, we are interested in probability of transition from the healthy (oscillating R expression) attractor to the pathological where R is chronically over-expressed (e.g. *p*_21_ in the transition matrix $\prod $ where *i*=1 for pathological and *i*=2 for healthy attractor).
- *Therapeutic blockade of R*: Here, we simulate the effects of knocking out or strongly inhibiting R (e.g. using mifepristone) in our model as means of a therapy. In this case, we are interested in both the probability of treatment resistance or remaining in pathological state (e.g. *p*_11_) as well as treatment response prompting a return transition to the healthy regulatory regime (e.g. *p*_12_).

**Fig. 4:**
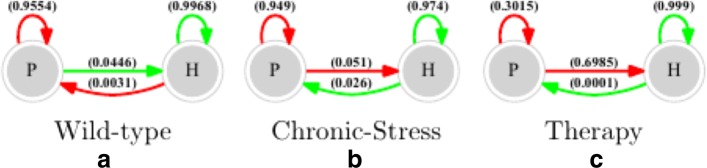
Mapping the Relative Resilience of a Regulatory Program. Graphical representation of the estimated probability of occurrence (State Transition Matrix) of state transitions resulting in a return to a given attractor versus an escape to the neighboring attractor. Markov Chain Monte Carlo simulations repeated 1000 times for each of the 36 possible initial states, were conducted with 5% random biological noise applied to an unperturbed HPA axis at rest (wild type) (**a**), to conditions of persistent environmental stress (**b**), and under therapeutic blockade of R (**c**). Results suggest that conditions of persistent stress facilitate the transition to a chronic HPA dysregulation while downregulation of R significantly destabilizes the latter favoring a return to normal HPA rhythm

Transition probabilities under random biological noise or decisional error *ε*=0.05 for each of these experiments is shown in Fig. 4. The probability of transition of the HPA axis from a healthy to a pathological regulatory regime is increased approximately tenfold under conditions of sustained external stress from 0.003 to 0.026. Should migration occur under these conditions, the probability of escaping this quite stable pathology remains roughly the same as in the undisturbed state at approximately 0.05. In Fig. 4c we show that the probability of an escape transition from this robust pathological steady state back to the healthy regulatory regime improves dramatically from 0.05 to 0.69 with the introduction of an R antagonist. In addition, the probability of relapse back into the pathological state falls by 2 orders of magnitude to 0.0001, making this transition extremely unlikely.

**Fig. 5:**
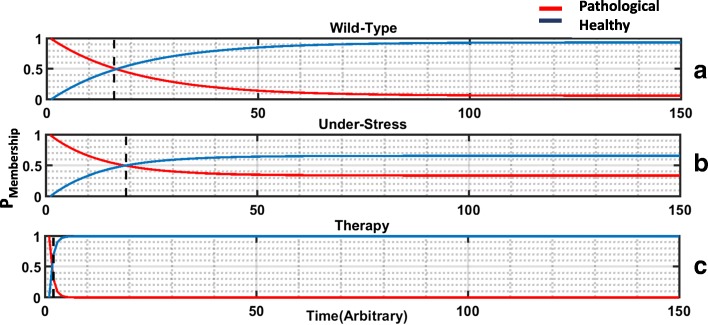
Simulating Escape from Pathological Dysfunction. Monte Carlo simulations of Markov Chains models tuned to the migration probabilities in each State Transition Matrix showing the expected evolution in time in the probability of continuing to occupy the pathological regime (red line) versus occupying the Healthy regime (blue line) assuming 5% random biological noise. Under conditions of persistent stress (**b**) any potential escape from pathological dysfunction is delayed (dotted vertical) compared to an unperturbed resting state (**a**). Conversely, with the introduction of a therapeutic blockade of R, a spontaneous escape from HPA dysregulation is virtually instantaneous (**c**). Time is plotted on an arbitrary scale and is measured in iterations

To illustrate this further, we applied the transition probability matrices computed in the previous step to a Markov Chain model and simulated the likelihood over time of escaping the pathological overexpression of R under conditions of undisturbed rest, chronic stress and therapeutic blockade of R. For each of these conditions, the Markov Chain model of each scenario was used to infer the average (number of time steps leading to a 50:50 chance of escaping the pathology and the resulting probabilities of relapse or conversely of remaining in the new healthy regime (Fig. 5). The results of the Markov Chain simulations show that the average number of time steps involved in transitioning from the pathological state to the healthy state are quite different for each scenario. As might be expected, if HPA axis dynamics currently adhere to the pathologic regulatory program P then the application of chronic stress serves to further galvanize this condition. Specifically, chronic stress would significantly delay a situation where the system might have a 50:50 chance of escape (almost one quarter). Moreover, the probability of remaining free of this pathology and maintaining a stable healthy state H never exceeds 70% (the Markov chain transition probabilities stabilizes in the middle figure). Conversely the introduction of a R antagonist almost immediately destabilizes the pathological attractor resulting in a rapid shift to the healthy regulatory regime H. Moreover, under this R blockade the probability of remaining resilient to stress and avoiding relapse is much higher, exceeding 90%.

## Discussion

### Logical Modeling and Parameterization

In this study, we employed a generalized discrete formalism in order to explain the complex cyclic bi-stable behavior of the HPA axis. By enforcing expected qualitative behaviors formally by requiring the requisite negative and positive regulatory feedback loops, we were able to recover two cyclic steady states that closely mimic the results of more complex ODE based simulations [7]. An alternative but similar methodology is also proposed in Devloo’s work [18], however the latter, focuses on the identification of steady states and not the parametrization of the supporting logic. Recently, other model checking techniques [19, 20] have also been proposed for identifying logic parameters efficiently. These methods rely on experimental time course measurements and do not accommodate qualitative behavior (e.g. bi-stability, cyclic attractors). It should be noted that we expect the combination of qualitative and quantitative results to yield a smaller and more accurate parameterization space commonly available to such models. Sedghamiz et al. [21] employs a Constraint Satisfaction (CS) technique along with biologically inspired cost functions to make the parameterization much more efficient. Indeed the small network model used here was selected as an example benchmark problem since the dynamics of the HPA axis have been well studied and a detailed conventional ODE model [7] was available as a comparator. However, this multi-valued logical model becomes more useful as the size of the network increases. First, the availability of comprehensive and detailed kinetic data becomes increasingly sparse as the networks become larger especially when these bridge across multiple physiological regulatory axes. In such cases the proposed framework allows one to continue exploring network dynamics by drawing the typically much broader knowledge of connectivity, for example receptor-ligand biochemistry. Secondly as might be expected, there exists an important computational advantage as the parameter search space for logical models is discrete, and thus significantly smaller. Our group recently reported the use of Constraint Programming to further enhance the scalability of model parameterization [21] and is applying this successfully in ongoing work to a number of well-studied problems, the largest of these consisting of 114 entities connected by 129 interactions describing gene regulation in dendritic cell differentiation [22]. Other studies have also shown the promising scalability of logical modeling with the help of model checking techniques and Answer Set Programming [19, 23].

### Complexity of attractor detection

Identification of attractors in this study is performed with a linear time modified Tarjan’s algorithm [24]. These methods have a time complexity of *O*(|*V*|+|*E*|), where *V* is the number of nodes and *E* number of edges in the state transition graph (STG). Accordingly, this time complexity is closely related to the type of the update scheme chosen. The STG generated under synchronous update is the smallest graph as each state has only one out degree, or successor state available. Therefore, in the case of a regulatory network with *n* nodes each taking *m* states, the STG has |*V*|=*m*^*n*^ nodes and |*E*|=*m*^*n*^ edges, yielding a time complexity of *O*(2*m*^*n*^). In contrast, an asynchronous update would typically generate a very large STG as a regulatory graph with *n* variables will at each transition have a maximum *n* successors or out-degrees. This equates to an architecture where |*V*|=*m*^*n*^ and |*E*|=*nm*^*n*^ resulting in a complexity of *O*(*m*^*n*^(1+*n*)). The priority class update has a time complexity that lies between these two extreme cases, namely *O*(2*m*^*n*^)≤*O*_*priority*_≤*O*(*m*^*n*^(1+*n*)). Consequently, traversing the STGs formed by the regulatory networks with more than 40 nodes might become intractable. There are methods that have employed symbolic representation of STG in order to search for attractors in a more efficient manner [22, 25]. However, application of these methods is limited to Boolean networks only. Recently, fast parallel algorithms [26] have been proposed for the detection of Strongly Connected Components (SCC) in large graphs. A modification of such algorithms might be used in order to more efficiently traverse large STGs.

### Context responsive remodeling of HPA axis behavior

Due to biological variability in the way environmental stressors are perceived and their severity assessed, we explored this interaction of an idealized stressor with the HPA axis in more depth. We identified multiple stable states that remained feasible as long as persistent stress was applied. In the absence of persistent stress these stress-potentiated states become dynamically unstable and resume their role as transient states in either the low-range or high-range cortisol oscillatory regimes. We showed that model parameters associated with increased sensitivity to external stress offered additional opportunity for transitioning into a stable regulatory state characterized by overexpression of the glucocorticoid receptor R.

Neural circuitry mediating sensitivity to perceived threat are reported to be a distinguishing component among several stress-induced illnesses [27] including PTSD where studies show hyperarousal as a consistent feature of this illness [28]. Accordingly, the predictions of this simple model align with results from animal studies suggesting that chronic stress leads to the persistent overexpression of the glucocorticoid receptor (R) [29]. The correspondingly low cortisol levels have been associated with metabolic mediation of stress-related disorders [30], including post-traumatic stress disorder (PTSD) [31] as well as fatiguing illnesses such as chronic fatigue syndrome [32]. Conversely persistently high cortisol levels have been associated with anxiety and major depressive disorders [33]. While it is recognized that differences in sensitivity to stress directly affect vulnerability to lasting HPA axis dysfunction, the adaptive states that exist in the presence of chronic stress and that may predispose to these conditions have not been well-studied. The simple model presented here predicts that in the case of stress-sensitive subjects a unique adaptive state exists characterized by high cortisol levels and elevated expression of R. Experimental validation of this marker co-expression pattern in, for example, a statistically significant segment of the hyper-aware subjects (new military recruits for example) would support the involvement in illness onset of these adaptive states. Such an assessment, potentially using a sub-maximal exercise challenge, could serve in the screening of candidates that might be exposed more frequently to such conditions, for example first responders. To further assess how easily accessed or entrenched these conditions might be, we also studied the model from a stochastic perspective by considering the effects of random biological noise included as a probability of a decisional error in the signaling mechanisms. Our simulations showed that under conditions promoting the over-expression of CRH (e.g. by chronic stress) the chance of falling into a stable state of pathological hypo-cortisolism increases. Conversely the model predicts that down-regulating R would highly increase the probability of transition to the alternate oscillatory steady state, essentially making the pathological persistent overexpression of R dynamically unstable. While stochastic simulations are well studied and modeled in Boolean networks, as far as we are aware, this has not been fully explored in multi-valued networks and we propose that this work is one such novel attempt. Indeed, in this work we extend the concept of probabilistic failure of regulatory function introduced by Garg et al. [34], and applied to Boolean networks, to the much more complex case of multi-valued logic.

Plasticity in stress response leading to context specific changes in the availability of specific HPA axis response programs have been observed in nature. For example, reduction in exposure to light, simulating seasonal shortening of daylight hours, resulted in increased corticosterone responses to restraint in mice, increased hippocampal glucocorticoid receptor expression, enhanced corticosterone negative feedback on the HPA axis, and increased sensitivity to dexamethasone suppression of corticosterone. Conversely, during periods of food scarcity resulting in caloric deficiency, Maniscalco and Rinaman report [35] that many physiological and behavioral responses to acute stress centrally mediated by the HPA axis are significantly attenuated such as anxiety and fearful behavior as well as normal stress-induced loss of appetite in favor of food foraging and intake behavior. They propose that this altered programming is due to reduced recruitment of A2 noradrenergic (PrRP+ A2) and hindbrain glucagon-like peptide (GLP-1) neurons, with correspondingly reduced signaling mid and forebrain targets. Indeed, Rabassa et al. [36] report that under conditions of chronic daily stress, acute response to a stressor resulted in a dampened expression of HPA axis markers such as ACTH further reinforcing the notion that alternate stable response programs can be induced by exogenous environmental factors. Such environmental remodeling of the attractor topology will also be affected by regulatory physiology adjacent to the HPA axis. For example, as with changes in metabolic status and its regulation by the hypothalamic-pituitary-thyroidal (HPT) axis, availability of specific stress response programs may be further modified by sex and regulation of the hypothalamic-pituitary-gonadal (HPG) axis [37].

As one might expect, adaptive changes in stress response programming will be similarly affected by pharmaceutical agents such as amphetamines which dampen the prototypic peripheral physiological response to stress and activation of the paraventricular nucleus (PVN) [38]. In this work, we propose a novel perspective on drug action, namely one where a pharmaceutical agent serves not to artificially maintain an otherwise unstable response but instead to render unstable an otherwise stable pathology e.g. a chronic regulatory imbalance. For example the hormone ghrelin has been reported to destabilize the chronic inflammatory cascade characteristic of osteoarthritis (OA) rendering this pathologic program unstable by rebalancing the interplay between Akt and NF- *κ*B signaling pathways [39]. Multi-target regulators such as BCG vaccine have been shown to impart protection against a number of autoimmune illnesses by activating anti-viral immune programming and in essence undermining the stability of antibody-mediated cascades [13, 14]. We extend this concept further by attempting to quantify the extent of this stability from the design and tuning of the regulatory circuitry itself, in essence describing the risk of onset or subsequent relapse in terms of the geography of a given attractor. Indeed, Gordon et al. [40], report that shifting topology of the hormonal regulatory environment during premenopausal transition may increase vulnerability to environmental psychosocial factors leading to heightened risk of depression. Likewise, although initial responses to first-line therapy are high in chronic myeloid leukemia (CML), this response dissipates within 2 years in approximately 25% of patients. Therapy eventually fails outright in up to 40% of patients [41] suggesting that while the desired response was indeed accessible, the corresponding attractor remained shallow enough to allow escape and relapse in a significant number of individuals. One could argue therefore that it is not sufficient to provide access to the correct regulatory program, it is also necessary to alter the landscape in a way that this attractor is sufficiently deep and resilient to biological variability.

## Conclusion

There is a growing appreciation for chronic diseases as the consequences of biological systems becoming trapped in abnormal steady states. In this work we use concepts initially developed by Thomas [42–44] and recently reviewed in Abou-Jaoudé et al. [45] to rigorously describe the function of regulatory networks in a discrete logic formalism that requires only minimal parameter fitting. This formalism is used to design and implement methods that combine computational efficiency with biological fidelity in capturing the multi-stable oscillatory modes of a major physiological regulator, namely the hypothalamic-pituitary-adrenal or HPA axis. Specifically, we enforce the generic properties of elementary feedback circuits, namely oscillatory dynamics and multi-stability, on a general model in order to efficiently tune parameter values and identify stable regulatory modes. This simple model of the HPA axis based on a segmented binary logic separated by activation thresholds reproduced the same complex dynamic behavior as that supported by a much more sophisticated set of ODE recently proposed by Kim et al. [7]. Importantly in this work we we extend the priority class asynchronous updating scheme of Faure et al. [46] by adding a memory of recent update, essentially reinforcing delayed activation. It is important to note that inclusion of a state transition memory in this update scheme was necessary in order to recover complex bi-stable oscillatory behavior. The simplicity of our implementation of the Thomas conceptual framework is significant not only because it offers a compact parameter space but more importantly because this framework makes it possible to explore the behavior of much broader physiology. For example, systems such as the HPA axis can be cast in a much more comprehensive context, one that accounts for interactions with neighboring metabolic, sex hormone, immune and central nervous system regulators [47, 48] even when little is known about the dynamics linking these different domains.

Indeed, the multi-valued logical formalism employed in this study has several important advantages over conventional Boolean networks. These include the increased discrete state resolution and related support of concentration-dependent actions, both which were needed here to reproduce the oscillatory split-range dynamics of the model HPA axis. Furthermore, the logical K values employed with this formalism can express all the possible combinatorial effects of co-factors in a more efficient and compact form than only using simple logical keywords such as AND, OR and NOT. Nonetheless, the main limitation of discrete logic-based models (binary and multi-valued) is that they express time as the number sequential state transition events. In general therefore, ODE models are more quantitative and accurate if proper parameters for the model are known, while the qualitative logical modeling techniques such as this are better suited for scenarios where kinetic parameters are difficult or impossible to accurately estimate.

In addition to reproducing the behavior of the HPA axis in isolation, this work graphically demonstrates how external factors may modify the overall regulatory circuit and shift the corresponding state transition landscape. Such modifications may make typically unavailable attractors suddenly available. Indeed, our analysis of this simple model of HPA biology predicts specific stress-potentiated stable states that straddle both basins of attraction offering a tentative mechanistic model for the potential course of onset in chronic HPA dysregulation. Importantly we link these potential avenues of onset to changes in the sensitivity to perceived stressful events, showing that changes in this biology may affect vulnerability by making available under stress additional states that occupy the opposite attractor. This same shift in regulatory landscape may also apply in the case of host-pathogen interactions [49] or therapies where drug effects may make available attractors consisting of pathogenic side effects. As a result, we contend in this work, that in addition to direct pharmacological side-effects, drugs and treatment programs should also be assessed in terms of the attractors that they may inadvertently render accessible. This accessibility will also be mediated by environmental effects and other outside influences which as additional components of the regulatory circuit may alter the geometry of these regulatory traps making them broader still. While the concept of remodeling of an attractor landscape by an externally applied stressor or pharmaceutical agent may not in and of itself novel [50–52], one could argue that such concepts are still not widely applied in the design of intervention strategies and its operationalization remains an area of ongoing research. Additionally we propose that such investigations have so far been restricted to binary networks and that the use of multi-valued logic in exploring these phenomena is, to our knowledge, novel.

We chose to study the HPA axis since it has been thoroughly studied, thus providing a well-established ground truth against which we could compare our predicted model parameter sets. Elevated levels of glucocorticoid receptors play a key role in many diseases related to the HPA axis [53]. A prominent example is Gulf War Illness (GWI), where the heightened and sustained stress of a combat environment and its continued stimulation of the HPA axis may have increased the vulnerability of personnel to environmental exposures and facilitated the migration to hyper responsive neuroinflammatory response program [54]. In this like in many treatment resistant conditions, static single target interventions have proven largely ineffective [3] supporting the notion that designing an effective escape trajectory may require a better knowledge of the regulatory dynamics at play. Knowledge such as this would inform not only on the best physiologic regulatory target (e.g. R) but would also inform on the best context (i.e. instantaneous state of co-regulators) in which to apply an intervention. Depending on this context, the recovery may follow a longer or shorter trajectory or fail outright. Perhaps more importantly still, this strategy of externally reshaping the attractor landscape might also be applied pro-actively towards developing protective strategies directed at increasing the resilience of a healthy regulatory program in anticipation of a stressor. In addition to recapitulating the known behavior of the HPA axis, we predicted that chronic, sustained stress would remodel the attractor landscape. One of the newly-available steady states is a unique attractor characterized by chronic overexpression of cortisol and the glucocorticoid receptor, which could increase sensitivity to further dysregulation of the HPA axis. Pre-emptively dampening the expression of glucocorticoid receptor R may offer an attractive candidate strategy for reducing the vulnerability of civilian first-responders or military personnel in combat to the stress-mediated onset of immune and endocrine dysfunction.

## Methods

### System of ordinary differential equations for HPA axis

Kim et al. [7] proposed the following system of delay differential equations to describe behavior of the HPA axis:

1 $$\begin{array}{*{20}l}  \frac{{\mathrm{d}C_{s}}}{\mathrm{d}{T}} &= \frac{C_{\infty}(O) - C_{s}}{T_{c}}. \end{array} $$

2 $$\begin{array}{*{20}l}  \frac{\mathrm{d}C}{\mathrm{d}T} &= p_{c}I(t)h(C_{s})g_{c}(C) - d_{c}C. \end{array} $$

3 $$\begin{array}{*{20}l}  \frac{\mathrm{d}A}{\mathrm{d}T} &= p_{A}C\left(\frac{K_{A}}{K_{A} + OR}\right) - d_{A}A. \end{array} $$

4 $$\begin{array}{*{20}l}  \frac{\mathrm{d}O}{\mathrm{d}T} &= P_{O}A(T-T_{d})-d_{o}O. \end{array} $$

5 $$\begin{array}{*{20}l}  \frac{\mathrm{d}R}{\mathrm{d}T} &= P_{R}\left(1-\frac{\mu_{R}k^{2}_{R}}{k^{2}_{R} + (OR)^{2}}\right) - d_{R}R. \end{array} $$

Where *C*_*s*_, *C*, *A*, *O*, and *R* are concentrations of synthesized *CRH*, released *CRH*, *ACTH*, *CORT* and the glucocorticoid receptor *R* respectively. For more details about these parameters and the governing equations see [7]. These equations translate into the HPA regulatory network shown graphically in Fig. 1a. Due to the complexity of parameter tuning and the often-limited availability of parameter estimates supporting ODE sets such as these, we look to the generalized formalism introduced by Thomas [12]. We use the latter to derive an equivalent set of discrete equations that also capture HPA behavior but draw mainly on the connectivity of the regulatory network and require only minimal parameter support.

### Discrete generalized formalism

In order to facilitate the analysis of discrete networks, we borrow the notation of piecewise linear differential equations from Snousi et al. [55]:

6 $$\begin{array}{@{}rcl@{}}  \frac{\mathrm{d}x_{i}}{\mathrm{d}T} = k_{i\emptyset} + \sum{k_{ij}S^{\alpha_{ij}}(x_{j},\theta_{ij})}-k_{i}x_{i}. \end{array} $$

Where *x*_*i*_ is the state of variable *i* in the network, *k*_*i∅*_ is an independent term representing the basal value of each variable (biologically we assume that there might always exist a basal concentration of species *i*), *α*_*ij*_∈{+,−} is the interaction sign (activation, inhibition respectively) from *j* to *i*, *θ*_*ij*_ is the threshold above which the interaction from node *j* to *i* is active, $\phantom {\dot {i}\!}S^{\alpha _{ij}}(x_{j},\theta _{ij})$ is a binary function computing whether the state of node *j* is above the activation threshold for this interaction, and finally *k*_*i*_>0 is a decay term for state variable *i*.

7 $$\begin{array}{@{}rcl@{}} S^{\alpha_{ij}}(x_{j},\theta_{ij}) = 1\leftrightarrow \left\{\begin{array}{ll} \alpha_{ij} &= + \land (x_{j} \geq \theta_{ij}),\\ \alpha_{ij} &= - \land (x_{j} < \theta_{ij}). \end{array}\right. \end{array} $$

Using this notation, the regulatory network might be formulated as a signed, weighted and directed graph with *N* vertices and *E* edges; where an edge (*j,i*,*α*_*ij*_,*θ*_*ij*_) states that the change in the expression level of variable *x*_*i*_ depends on the concentration of *x*_*j*_ (i.e. when *k*_*ij*_≠0) if it is above the threshold *θ*_*ij*_. The steady state $x_{i}^{0}$ of this equation is the solution when *x*_*i*_*T*=0. Therefore,

8 $$\begin{array}{@{}rcl@{}}  x_{i}^{0} = \frac{1}{k_{i}} \bigg[ k_{i\emptyset}+\sum{k_{ij}S^{\alpha_{ij}}(x_{j},\theta_{ij})} \bigg]. \end{array} $$

Applying a simple discretization operator results in,

9 $$\begin{array}{@{}rcl@{}}  D\left(x_{i}^{0}\right)= D\left(\frac{k_{i\emptyset}}{k_{i}}\right)+D\left(\sum{\frac{k_{ij}}{k_{i}}}S^{\alpha_{ij}}(x_{j},\theta_{ij})\right). \end{array} $$

If we denote the *ratio of synthesis to decay* kinetics with $K = \frac {k_{i\{\emptyset,ij\}}}{k_{i}}$, we will have,

10 $$\begin{array}{@{}rcl@{}} D\left(x_{i}^{0}\right)= D(K_{i\emptyset})+D\bigg(\sum{K_{ij}}S^{\alpha_{ij}}(x_{j},\theta_{ij})\bigg). \end{array} $$

Consequently, the logical K parameters in the general formalism simply are the ratios of the synthesis to decay kinetics. After deriving the fundamentals of discrete formalism, we are ready to introduce an image function that is a discrete approximation of Hill-type ODEs. It can be shown that Eq. 10 might be written as Eq. 11 and solved iteratively (see [56] for more details),

11 $$\begin{array}{@{}rcl@{}} y_{i}\! =\! \sum_{I \subseteq q(i)}K_{i.I}\left[\!\prod_{j\in I}S^{u_{ij}}(x_{j},w_{ij})\prod_{j\in q(i)\backslash I} \left(1 - S^{u_{ij}}(x_{j},w_{ij})\right) \!\right]\!.  \end{array} $$

Where *q*(*i*) is the in-degree set of component *i*. **Y**=[*y*_1_,…,*y*_*N*_] is called the *image* vector of graph *G* with *N* components given its current state vector **X**=[*x*_1_,…,*x*_*N*_]. The image function simply states that when there is no active interaction modulating *x*_*i*_, the image is equal to its basal value *K*_*i∅*_=*D*(*k*_*i∅*_) and when more than one interaction is applied concurrently, the image is equal to their joint logical parameter (e.g. *K*_1.12_=*D*(*k*_11_+*k*_12_) when both variable 1 and 2 are modulating variable 1). According to Eq. 11, the corresponding discrete approximate ODE model of the HPA axis (see Eqs. 2 – 5) can be expressed in a generalized discrete form (See Additional file 6 for the derivation of these equations).

We apply the updating scheme described in the next section to the image function in order to simulate the evolution in time of the discrete HPA axis network. This consists of establishing a protocol for scheduling the transition of each state variable (e.g. network node) towards the target state computed by its corresponding image function. These state transitions towards the next target state can be scheduled to occur in synchrony or according to a physiologically plausible sequence based on an assigned priority. Thomas et al. [12] was one of the first researchers to suggest that since cells/genes do not necessarily change their transcription level simultaneously, a complete stochastic and asynchronous update might be used. However numerous examples exist, including determination of cell fate, showing a balance between order and stochasticity in biology [57]. Accordingly, in this work, we compare three different types of state transition schemes; *fully synchronous*, *asynchronous* and a *hybrid approach* based on assignment to a priority class with a memory of previous states.

### Update schemes

In this section, we briefly introduce the update schemes employed in this study. First, we define a *tendency* function for the network as;

12 $$\begin{array}{@{}rcl@{}} f(x_{i})^{t} = \left\{\begin{array}{lll} x_{i} + 1,& if~x_{i} < y_{i},\\ x_{i},& if~x_{i} = y_{i},\\ x_{i} - 1,& if~x_{i} > y_{i}. \end{array}\right.  \end{array} $$

Where **F**(*x*)^*t*^=(*f*_1_(*x*),*f*_2_(*x*),…,*f*_*n*_(*v*)) defines tendency of the network at time *t*. The tendency function determines the gradient of change in the concentration of entity *i* at time point *t*. This set of rules simply state that if the image function *y*_*i*_ of a variable is less (more) than its current state (*x*_*i*_), its successor tendency state (*f*(*x*_*i*_)^*t*^) is a single step decreased (increased). This definition is similar to Chaouiya et al. [56] and it ensures that a stepwise increase or decrease through each sequential intermediate state is enforced.

#### Fully synchronous update

One of the most common updating schemes is fully synchronous update of all state variable nodes. Under synchronous update, the successor of a state for a variable is computed based on the simultaneous update of all variables in the network. Therefore, the transition function is simply defined by taking the conjunction between all of the variables,

13 $$\begin{array}{@{}rcl@{}} T_{i}\left(\mathbf{F(x)^{t}},\mathbf{x^{t+1}}\right) = \left(x^{t+1}_{i} \leftrightarrow f(x_{i})^{t}\right). \end{array} $$

14 $$\begin{array}{@{}rcl@{}} \mathbf{T_{Syn}}\left(\mathbf{F(x)^{t}},\mathbf{x^{t+1}}\right) = \bigwedge_{i=1}^{N} T_{i}\left(\mathbf{F(x)^{t}},\mathbf{x^{t+1}}\right). \end{array} $$

#### Fully asynchronous update

A fully asynchronous update allows only a single variable at a time in the network to be updated to its successor state. As a result, a given state might have more than one possible successor state [22]. Since only one node changes its expression level at a time, we need to enforce,

15 $$\begin{array}{@{}rcl@{}} T_{i}\left(\mathbf{F(x)^{t}},\mathbf{x^{t+1}}\right) = \left(x^{t+1}_{i} \leftrightarrow f(x_{i})^{t}\right)\wedge \bigwedge_{j \neq i}\left(x^{t+1}_{j} \leftrightarrow x_{j}\right).  \end{array} $$

16 $$\begin{array}{@{}rcl@{}} \mathbf{T_{Asy}}\left(\mathbf{F(x)^{t}},\mathbf{x^{t+1}}\right) = \bigvee_{i=1}^{N} T_{i}\left(\mathbf{F(x)^{t}},\mathbf{x^{t+1}}\right). \end{array} $$

Note that taking the disjunction among the nodes assigns a uniform probability of change to each node and causes stochasticity contrary to the synchronous case which is deterministic.

#### Priority class with memory

Synchronous update has been criticized [12, 58] for its oversimplification of biology, since cell abundance or gene expression do not rigidly change in exact unison according to a master clock. On the other hand, it has been argued [58, 59] that asynchronous update is excessively random and it might be an exaggeration of the biological noise. Therefore, in this study, we introduce a strategy based on assignment to a priority class with longitudinal memory where each class (e.g. variable) is associated with a delay counter and a memory. This update scheme shares a few criteria with asynchronous update but its transition function is slightly different. The criteria for this update are:

- Only one variable may make a transition at each iteration (see Eq. 15)
- The variable with minimum residual memory is updated first; The memory variable for entity *i* ($m_{x_{i}}$) is initialized as a default non-zero positive integer *d*_*i*_ and updated based on the rules below:
  17 $$\begin{array}{@{}rcl@{}} F1_{c_{i}} = \left(y_{i}^{t-1} \oplus y_{i}\right). \end{array} $$
  18 $$\begin{array}{@{}rcl@{}} F2_{c_{i}} = \left(x_{i}^{t-1} \leftrightarrow x_{i}\right). \end{array} $$
  19 $$\begin{array}{@{}rcl@{}} m_{x_{i}}= \left\{\begin{array}{ll} min(d_{i},m_{x_{i}} + 1)& \text{if } F1_{c_{i}} \wedge F2_{c_{i}}, \\ max(1,m_{x_{i}} - 1)& \text{if } \neg F1_{c_{i}} \wedge F2_{c_{i}}, \\ d_{i} & \text{if} \neg F2_{c_{i}}. \end{array}\right.  \end{array} $$
  Note that *x*_*i*_, *y*_*i*_, $x_{i}^{t-1}$ and $y_{i}^{t-1}$ denote *current* state, image, *previous* state and image respectively. $F1_{c_{i}}$ flag is *true* if the current and previous image functions for node *i* are in *agreement* (either both are a command to increase or decrease in expression). $F2_{c_{i}}$ is *true* if the state of entity *i*, *x*_*i*_ does not change its expression in a transition from iteration *t*−1 to *t*. Finally, a flag function is computed to check whether residual memory $m_{x_{i}}$ is the minimum in the network:
  20 $$\begin{array}{@{}rcl@{}} F3_{c_{i}} = \left(\bigwedge_{j\neq i} m_{x_{i}} \leq m_{x_{j}}\right). \end{array} $$
  $F3_{c_{i}}$ is *false* if there exists at least one variable *x*_*j*_ that has a residual memory smaller than $m_{x_{i}}$. The transition for *x*_*i*_ is then defined as,
  21 $$ {{}\begin{aligned} T_{i}&\left(\mathbf{F(x)^{t}},\mathbf{x^{t+1}}\right)\\& = F3_{c_{i}}\wedge \left(x^{t+1}_{i} \leftrightarrow f(x_{i})^{t}\right) \wedge \bigwedge_{j \neq i}\left(x^{t+1}_{j} \leftrightarrow x_{j}\right)\!.  \end{aligned}}  $$
  22 $$\begin{array}{@{}rcl@{}} \mathbf{T_{pri}}\left(\mathbf{F(x)^{t}},\mathbf{x^{t+1}}\right) = \bigvee_{i=1}^{N} T_{i}\left(\mathbf{F(x)^{t}},\mathbf{x^{t+1}}\right). \end{array} $$

Priority class with memory is an extension of the basic priority class scheme employed by Fauré et al. [59] that supports transitions across the various timescales that govern HPA axis dynamics and where more sophisticated delayed differential equation models would typically be needed.

### Interactive parameter tuning algorithm

**Fig. 6:**
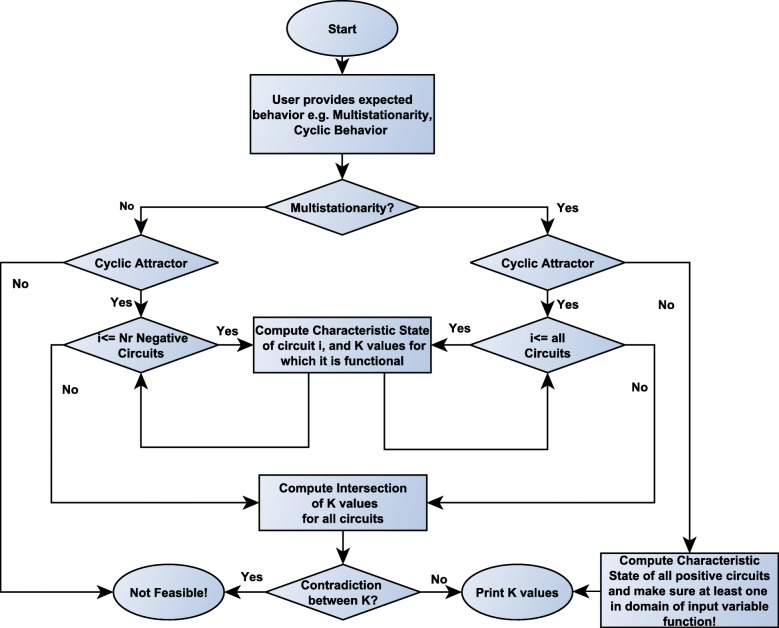
Algorithm Outline. Diagram describing the procedural logic linking the different algorithmic unit steps involved in the parameter estimation of activation threshold values *θ* and contextual logic weights K used in computing the state transition image for the system

Thomas [43, 44] was the first to study the cyclic attractors in terms of qualitative models. The author referred to these cyclic attractors as *singular* states and to stationary point or node steady states as *regular* attractors. The singular states correspond directly to feedback loops in the regulatory graph. Specifically, the cyclic attractors are associated with the elementary circuits, i.e. closed loop feedback circuits, that have negative parity in the regulatory networks. Snoussi [55] derived a set of in-equalities in order to better describe *singular* steady states. Singular steady states are also studied as trap-spaces in [60, 61]. Inspired from the same ideas, we have developed an algorithm that ensures multi-stability and existence of cyclic attractors. For a detailed overview and derivation of our proposed method see Additional file 1. Our approach is able to find a set of logical values that guarantee the existence of cyclic steady states(see Fig. 6 for the work-flow).

### Identification of attractors

In order to traverse the State Transition Graph (STG) (e.g. Fig. 1b) associated with a regulatory network, we employ a variant of Tarjan’s algorithm [24] in order to search for both regular (stationary point) and singular (cyclic) attractors (see Additional file 7 for more details).

### Stochasticity analysis

Biological *functions* are mainly grouped into: *barely, moderately* and *highly* stochastic. Examples of such functions are Ribosome (barely stochastic), Transcription (moderately stochastic) and Scaffolding complexes (completely stochastic). Stochasticity is modeled similar to *Chemical Master Equation* (CME) approach [62] where departure of an entity from its prescribed order of instantiation depends on the activity of other nodes in that instant of time. This biologically inspired criteria helps modeling robustness naturally [34]. We extend these ideas from binary simulations to multivalued regulatory models where entities have higher degrees of freedom.

#### Stochasticity in functions

As mentioned earlier the levels of stochasticity is different depending on the entities and functions being modeled. Therefore, a probability of fault in function (**ε**) is introduced that determines the confidence on occurrence of a biological function. A biological function might behave stochastically only when it is dynamically active and receives signals from other entities. For instance, a gene might change its expression only when it receives a signal to do so and there is a chance of fault in this purpose. Therefore, among all the entities in the model only those nodes that are receiving signal might exhibit a faulty behavior. Then, a Boolean vector **B**={*β*_1_,…,*β*_*v*_} with cardinality equal to the number of variables in the model is defined that determines whether at time point *t* there is at least one active interaction on node *v*.

23 $$\begin{array}{@{}rcl@{}} \beta_{v} = 1 \leftrightarrow \{w \in V | (w,v) \in E \wedge \big(x_{w} \geq \theta_{vw}\big) \} :\neq \emptyset \end{array} $$

Then among non-zero bits of **B***only* one is selected with a uniform probability of: 
24

Where  is the cardinality. Then a constant probability of error *ε* is used for which the selected entity *β*_*v*_ might disobey its expected image function order. Note that this is a single fault model and that is why only one fault at a time is allowed in the simulations. Finally, the probability that the image function associated with *β*_*v*_ has a fault (e.g. *P*(*β*_*v*_=1)) is :

25 $$\begin{array}{@{}rcl@{}} P(\beta_{v}=1) = \beta_{v}.\epsilon \end{array} $$

The faulty tendency function is defined as;

26 $$ \begin{aligned} \widehat{f(x_{v})}^{t} = \left\{\begin{array}{ll} x_{v},& if~(x_{v} < y_{v}) \vee (x_{v} > y_{v}),\\ x_{v}+1,& if~x_{v} = 0,\\ x_{v}-1,& if~x_{v} = \rho_{v},\\ (x_{v}-1) \vee (x_{v}+1),& if~(x_{v} = y_{v}) \wedge (x_{v} \neq \{0,\rho_{v}\}). \end{array}\right. \end{aligned}  $$

Where *ρ*_*v*_ is the max expression level of node *v* (compare this to Eq. 12). In order to extend the analysis into a discrete stochastic simulation, first the basins of attractions corresponding to each attractor $(basin_{ss_{i}} \in SS)$ are computed under *ε*=0 probability of fault. This is simply the set of all backward reachable sets to each attractor building a destination map for each node in STG. Then, a probability of fault *ε*>0 is introduced and for each member of the STG a set of monte carlo simulations are performed for a high number of times (e.g. 1000). The number of times that a state *s* belonging to basin of attractor *i* is mapped to attractor *ss*_*j*_ is then computed. Therefore, a transition matrix for the attractors of the model are formed as:

27 $$\begin{array}{@{}rcl@{}} \pi_{ij} = P(s \in basin_{ss_{i}}|s \in basin_{ss_{j}}). \end{array} $$

Using a time homogenous Markov Chain (MC), one can predict the transition probability of attractors, given the initial state as;

28 $$\begin{array}{@{}rcl@{}} P_{ss}(t+1) = \prod P_{ss}(t) \end{array} $$

Where $\prod $ is the transition matrix and *P*_*ss*_(*t*) the transition distribution at time point *t*.

## Additional files

Additional file 1 Interactive Parameter Tuning Algorithm. Describes the parameter identification algorithm and pseudo-code. (PDF 300 kb)

Additional file 2 Stress Response Parameterization. Parameterization of stress response. (PDF 332 kb)

Additional file 3 Asynchronous Simulation. Asynchronous update of the network contains two complex cyclic attractors. Since under asynchronous update each state might have more than two successors several state nodes in the graph present with multiple out-bound edges. (PDF 52 kb)

Additional file 4 Synchronous Simulation. Simulations with synchronous update where each node has only one successor state. It is well known that synchronous update can result in spurious cycles [12]. (PDF 36 kb)

Additional file 5 Priority Memory Update. Simulations in priority class with memory. CORT is placed in a second priority class update and the rest of the parameters in the model have a higher priority and frequency of update. (PDF 24 kb)

Additional file 6 Discrete Mathematical Equations Governing HPA axis. Details the derivation of discrete equations employed for the simulation of HPA axis. (PDF 132 kb)

Additional file 7 Algorithms for Computation of Attractors. Pseudo-code and algorithm for computation of attractors. (PDF 151 kb)

## Abbreviations

ACTH: Adrenocorticotropin Hormone
CFS: Chronic Fatigue Syndrome
CME: Chemical Master Equation
CNS: Central Nervous System
CRH: Corticotropin Releasing Hormone
DDE: Delay Differential Equations
GWI: Gulf War Illness
HPA: Hypothalamic-Pituitary-Adrenal
HPT: Hypothalamic-Pituitary Thyroidal
ODE: Ordinary Differential Equations
PA: Pituitary Gland
STG: State Transition Graph
