# Supplementary material for: High-fidelity discrete modeling of the HPA axis: a study of regulatory plasticity in biology
Source: BMC Syst Biol. 2018 Jul 17;12:76. doi: 10.1186/s12918-018-0599-1 (PMC6050677; doi:10.1186/s12918-018-0599-1)
Supplement: Supplementary file 1 — Interactive Parameter Tuning Algorithm. Describes the parameter identification algorithm and pseudo-code. (PDF 300 kb) [file 12918_2018_599_MOESM1_ESM.pdf]

Additional File 2

Hooman Sedghamiz

December 7, 2017

Interactive Parameter Tuning Algorithm

Elementary Circuit

Definition 1. Given a regulatory graph G, a path in G is a sequence of vertices $P_{vu} = (v = v_1, v_2, \cdots, v_k = u)$ such that $(v_i, v_{i+1}) \in E$ for $1 \leq i < k$. A *circuit* is a path in which the first and last vertices are identical. A circuit is *elementary* if no vertex appears twice [2].
In the regulatory networks, we deal with elementary circuits. We will see that a regulatory network cannot have a cyclic attractor if it does not contain at least one elementary circuit.

Parity of Circuit

Definition 2. The sign of an elementary circuit is *positive* (*negative*) *iff* it has *even* (*odd*) number of edges with *negative* parity.

Characteristic State

Definition 3. Let $A = (v = v_1, v_2, \cdots, v_1)$ be an elementary circuit in the regulatory graph G. The characteristic state C of circuit A is defined as the state in which each vertex of A is located at its corresponding outdegree threshold value (e.g. θ_{12}). Formally,

$$\forall k \in \{1, 2, \cdots, n\}, C_{i_k} = \theta_{i_{k+1}, i_k}. \tag{1}$$

Lemma 1. Given a regulatory graph G, it contains a cyclic attractor *iff* it has at least a *functional* elementary circuit with *negative* parity[1]. For a variable x_i in the network to be a part of a cyclic attractor (to oscillate), it should be necessarily part of a functional elementary circuit (be characteristic state) with a negative parity otherwise it has a fixed state (does not oscillate);

$$\begin{cases} y_i = x_i & \forall x_i \in Z_r \\ y_{max_j} < \theta_j^{\lambda} \leq y_{min_j} & \forall x_j \in Z_s \end{cases} \tag{2}$$

[1] Or union of disjoint circuits with negative parity. Two circuits are disjoint if they have no node in common.

Where y_{max_j} and y_{min_j} are minimal and maximal images of node j. Z_r and Z_s is defined to be the set of regular and singular states in the network (See Example 1). θ_j^λ (θ_{j_{k+1},j_k}) defines the threshold of action for node j in the circuit.

Proof . Snoussi [1] in the 3rd section of his article formally introduces and shows the necessary conditions for cyclic attractors and their relation to elementary circuits in a regulatory graph. The way that logical parameters (K values) are set determines which circuits in the network are functional.

Example 1 . In HPA axis, there are three isolated circuits and one union of disjoint circuits;

- ACTH↪CORT⊣CRH↪ACTH[2]. As the number of negative edges is 1, so the parity of this circuit is negative and can generate a cyclic attractor in the domain of its members (CRH, ACTH and CORT).
- ACTH↪CORT↪R⊣ACTH. As the number of negative edges is 1, so the parity of this circuit is negative and can generate a cyclic attractor in the domain of its members (ACTH, CORT and R).
- R↪R which is a self-interacting circuit. As it has 0 negative edge, then it is a positive circuit. If the K values are set in a manner that this circuit is functional, the network necessarily has more than one stable state (can be node or cyclic depending whether any other negative circuit is functional simultaneously).
- (ACTH↪CORT⊣CRH↪ACTH)∧(R↪R) which is a union of two disjoint circuits and since it contains all of the variables in the network, by some authors [3] it is also called *nucleus* and its sign is defined as $(-1)^{p+1}$, where p is the number of positive circuits in the *nucleus*.

Based on Lemma 1, the variables belonging to the negative circuits are able to oscillate. For example, in circuit *"ACTH↪CORT⊣CRH↪ACTH"*, nodes ACTH, CORT and CRH are able to oscillate and R stays stationary. This is often denoted as the characteristic state of a circuit and symbolically written as $[\theta^{-1}\theta^{1}\theta^{1}*]$[3]. The nodes that belong to the circuit have been replaced by their threshold of action (θ_{ij}) and non-loop nodes (here node 4) can take any value in their maximum transcription range but will stay stationary (constant in the whole cycle).

Snoussi [1] proposed a simple recipe in order to efficiently set the logical values in a way that an isolated negative circuit is functional (therefore, creates a cyclic attractor). He proposed to limit the images of maximum and minimum adjacent states of the characteristic state of the circuit according to their threshold of action (θ_j^λ, see Eq 3). The maximal state is simply computed by assigning the threshold of arcs in the circuit and for the minimal state a unit below the threshold of arcs if the parity of the edge is positive and vice versa if negative. For instance, in circuit *"ACTH↪CORT⊣CRH↪ACTH"* (with characteristic state of $[\theta^{-1}\theta^{1}\theta^{1}*]$),

[2]The "↪" and "⊣" symbols stand for activation and inhibition respectively.

[3]The first three variables can oscillate between 0 to 1 and last should stay constant

two adjacent states are [011*] (maximal) and [100*] (minimal). Then, one needs to ensure the following system of equation holds;

$$
\begin{cases}
y_4 = x_4 \\
F([100*])_1 < 1 \leq F([011*])_1 \\
F([100*])_2 < 1 \leq F([011*])_2 \\
F([100*])_3 < 1 \leq F([011*])_3
\end{cases}
\tag{3}
$$

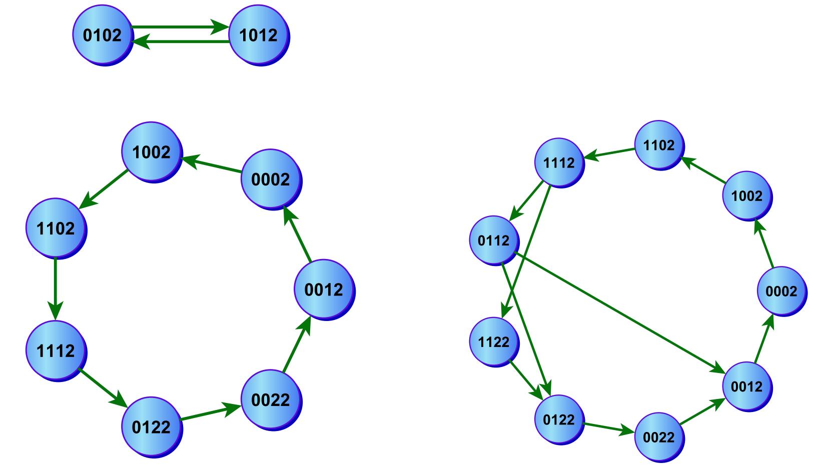

(a) Attractor shape under synchronous update

(b) Attractor shape under asynchronous update

Figure 1: Treating the steady trap space $[\theta^{-1}\theta^{1}\theta^{1}2]$ under Synch and Asynch update schemes leads to different shapes of attractors. However, regardless of the updating scheme in both attractors the first three parameters oscillate and the last is fixed at 2.

Note that each arc belonging to circuit "$ACTH \rightarrow CORT \dashv CRH \rightarrow ACTH$" has a threshold of 1 that it is why all of the last three inequalities are around 1. $F[100*]_i$ is the minimum image for variable i computed by placing $[100*]$ in the image function (see Eq. 11 in the article for definition of image function). Also, note that solving Eq 3 is computationally very efficient as it is sufficient to just enumerate the last variable (or any non-circuit variable). One of the solutions to Eq. 3 (solved with Constraint Satisfaction) is $[\theta^{-1}\theta^{1}\theta^{1}2]$ and there are 47 different combinations of K values satisfying it. We use a notion of *trap space* for such symbolic steady states (e.g. $[\theta^{-1}\theta^{1}\theta^{1}2]$). Any set among this 47 sets of K values is able to generate a trap space in which there is no trajectory escaping and the first three parameters oscillate and the last variable is fixed at state of 2.
After finding this trap space one can apply different updating rules in order to observe the precise shape of an attractor. For instance, $[\theta^{-1}\theta^{1}\theta^{1}2]$ gives rise to two different attractors illustrated in Fig 1 depending whether synchronous or asynchronous update is used.

In summary, our proposed algorithm performs the following steps:

I. Identify negative circuits in the regulatory graph. In fact linear algorithms ($O((n+e)(c+1))$ in time and $O(n+e)$ space, where there are n vertices, e edges, and c circuits) exist for fast identification of elementary circuits (See [2]). This algorithm scales well with graphs up to 1000 nodes.

II. Check the cyclic attractor in question ($\hat{X}$) for oscillating parameters and select all of the negative circuits that contain these parameters and solve for their state characteristic state (Form a similar system of equation as Eq 3).

III. After finding the trap space(s) apply a specific time update (e.g. Asynch or Synch) and discard those K values that do not match the exact shape of $\hat{X}$.

The pseudo code of the proposed method is shown in Algorithm 1. In a first step, we compute the characteristic state of the corresponding circuit (line 10) then we compute two states adjacent to the characteristic state whose images are maximal and minimal (line 7). The adjacent images are simply computed by assigning the same threshold θ_j^λ as the maximal image and a unit below the threshold as the minimal image if the parity of the edge is positive and vice versa if negative. The adjacent images are simply computed by assigning the same threshold as the maximal image and a unit below the threshold as the minimal image if the parity of the edge is positive and vice versa if negative. In a subsequent step, we use a backtracking algorithm to determine the values of K for which the characteristic state would lie between the adjacent images (line 15) for the nodes belonging to the elementary circuit while nodes outside this feedback loop are held at a constant state (e.g. R in the running example).

Algorithm 1: Computing logical parameters

```
input  : ADJ, adjacency list representing network
         Levels, maximum transcription level of each node
output: K, selected logical values
GenLogicalValue(ADJ)
begin
    K ← initial_k(ADJ), C ← findCycle(ADJ)
    for i = 1 : Levels.size() do
        A[i] ← ∅
    end
    CS ← // User selects the cycles for processing from C
    for i ← 1 : C.size() do
        CT ← C[i]
        for j = 1 : CT.size() do
            Lindex ← CT[j]) // compute characteristic state of the loop
            A[Lindex] ← Levels[j]
        end
        B ← compute adjacent states of A
        Backtrack and remove K values that are not located between B
    end
    check the unity of K for all the loops in query
    return K
end
initial_k(ADJ)
begin
    // Get number of nodes in regulatory graph
    N ← ADJ.size()
    for i ← 1 : N do
        K_i0 ← 0
        Indegree ← number of indegrees of i
        for j ← 1: 2^Indegree do
            K_ij ← {0 : Levels(i)}
        end
    end
end
```

References

[1] H. El Snoussi and R. Thomas. Logical identification of all steady states: The concept of feedback loop characteristic states. *Bulletin of Mathematical Biology*, 55(5):973–991, 1993.

[2] D. B. Johnson. Finding All the Elementary Circuits of a Directed Graph. *SIAM Journal on Computing*, 4(1):77–84, 1975.

[3] M. Kaufman, C. Soulé, and R. Thomas. A new necessary condition on interaction graphs for multistationarity. *Journal of Theoretical Biology*, 248(4):675–685, 2007.
