# Supplementary material for: High-fidelity discrete modeling of the HPA axis: a study of regulatory plasticity in biology
Source: BMC Syst Biol. 2018 Jul 17;12:76. doi: 10.1186/s12918-018-0599-1 (PMC6050677; doi:10.1186/s12918-018-0599-1)
Supplement: Supplementary file 2 — Stress Response Parameterization. Parameterization of stress response. (PDF 332 kb) [file 12918_2018_599_MOESM2_ESM.pdf]

Additional File 7

Stress Response

After adding stress as an external source node as an activator, based on the generalized formalism as CRH has a indegree of 2 (from Cort and Stress), there are 2^2 K values defining its new logical description. Intuitively,

$K_{1\emptyset}$ defines when stress is absent but Cort is inhibiting what the transcription level of CRH is,

K_{13} defines when Cort is *not* inhibiting and Stress is also absent what the transcription level of CRH is,

K_{15} defines when Cort is inhibiting and Stress is present what the transcription level of CRH is,

$K_{1.35}$ defines when Cort is *not* inhibiting and Stress is present what the transcription level of CRH is,

Therefore, this considers all the possible effects of stress on the network on CRH.

The rest of K values belonging to other nodes are kept the same during such analysis.

As there are 4 parameters each can take either 0 or 1 therefore, there are 16 possible parameterization in total, illustrated below. Note that some of the parameterizations intuitively make more sense such as the second one that implies, once stress is absent and Cort is inhibiting , CRH takes 0; similarly , once Cort is not inhibiting and stress is also absent CRH is also 0; also, once Cort is inhibiting and Stress is present still CRH takes 0; while once Cort is not inhibiting and Stress is present CRH over-express and tends toward 1.

<table>
<tr><th>Logical Configuration</th><th>Attractor under Priority update assumption (Stress = 1)</th><th>Attractor under Priority update assumption (Stress = 0)</th></tr>
<tr><td>$K_{1\emptyset} = 0$,
$K_{13} = 0$,
$K_{15} = 0$,
$K_{1.35} = 0$</td><td>

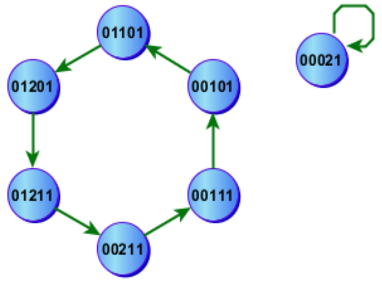

</td><td>

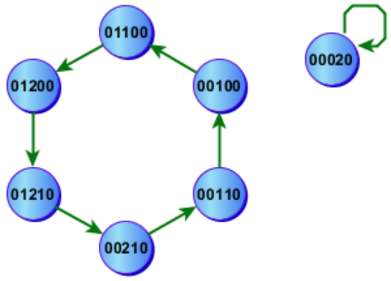

</td></tr>
</table>

$K_{1\emptyset} = 0$,<br>$K_{13} = 0$,<br>$K_{15} = 0$,<br>$K_{1.35} = 1$	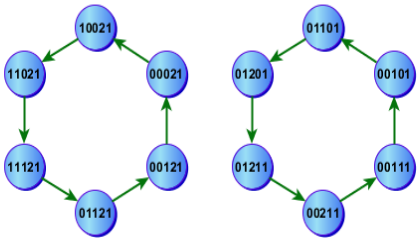	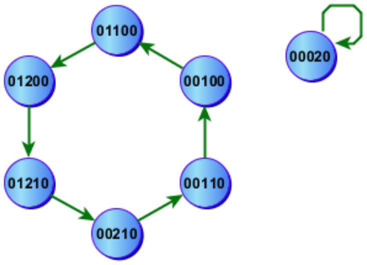
$K_{1\emptyset} = 0$,<br>$K_{13} = 0$,<br>$K_{15} = 1$,<br>$K_{1.35} = 0$	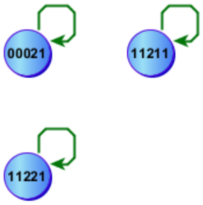	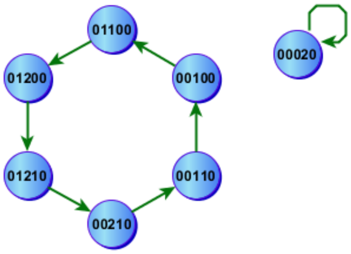
$K_{1\emptyset} = 0$,<br>$K_{13} = 0$,<br>$K_{15} = 1$,<br>$K_{1.35} = 1$	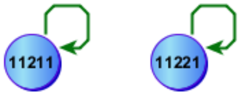	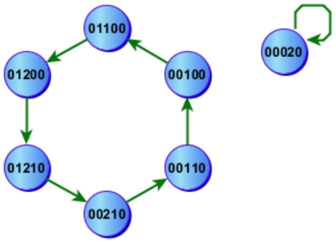
$K_{1\emptyset} = 0$,<br>$K_{13} = 1$,<br>$K_{15} = 0$,<br>$K_{1.35} = 0$	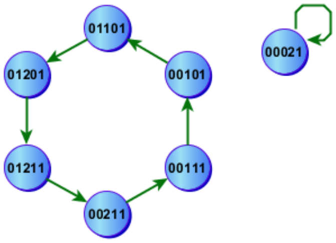	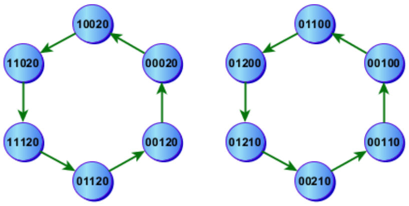
$K_{1\emptyset} = 0$,<br>$K_{13} = 1$,<br>$K_{15} = 0$,<br>$K_{1.35} = 1$	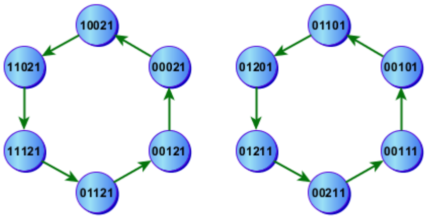	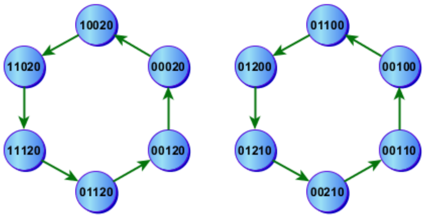
$K_{1\emptyset} = 0$,<br>$K_{13} = 1$,<br>$K_{15} = 1$,<br>$K_{1.35} = 0$	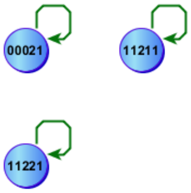	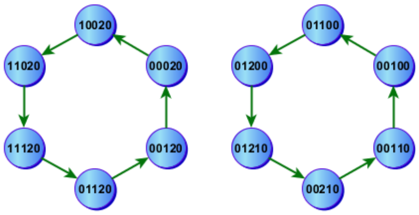

$K_{1\emptyset} = 0$, $K_{13} = 1$, $K_{15} = 1$, $K_{1.35} = 1$	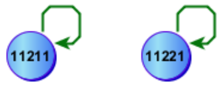	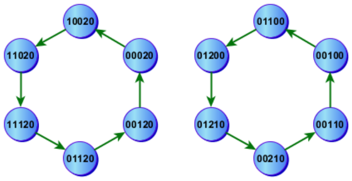
$K_{1\emptyset} = 1$, $K_{13} = 0$, $K_{15} = 0$, $K_{1.35} = 0$	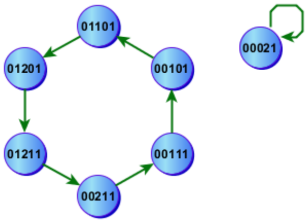	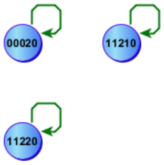
$K_{1\emptyset} = 1$, $K_{13} = 0$, $K_{15} = 0$, $K_{1.35} = 1$	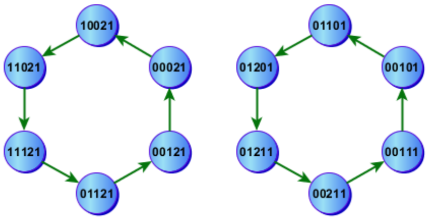	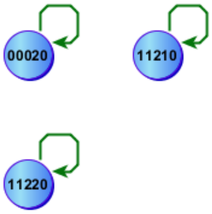
$K_{1\emptyset} = 1$, $K_{13} = 0$, $K_{15} = 1$, $K_{1.35} = 0$	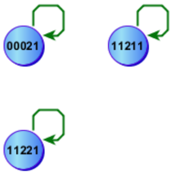	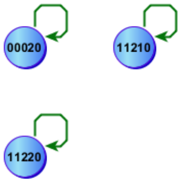
$K_{1\emptyset} = 1$, $K_{13} = 0$, $K_{15} = 1$, $K_{1.35} = 1$	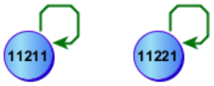	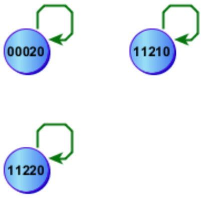
$K_{1\emptyset} = 1$, $K_{13} = 1$, $K_{15} = 0$, $K_{1.35} = 0$	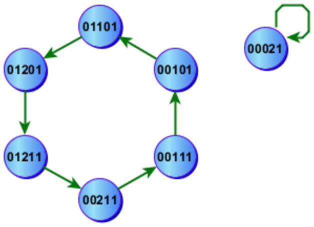	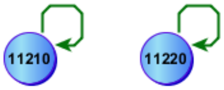
$K_{1\emptyset} = 1$, $K_{13} = 1$, $K_{15} = 0$, $K_{1.35} = 1$	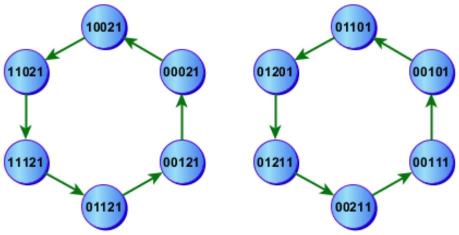	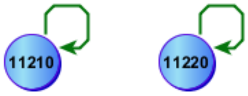

$K_{1\emptyset} = 1$,<br>$K_{13} = 1$,<br>$K_{15} = 1$,<br>$K_{1.35} = 0$	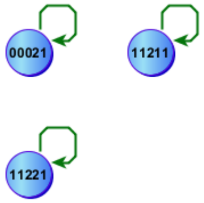	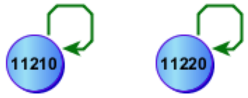
$K_{1\emptyset} = 1$,<br>$K_{13} = 1$,<br>$K_{15} = 1$,<br>$K_{1.35} = 1$	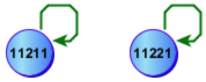	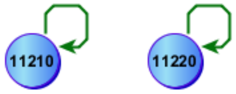
