# Supplementary figures and images for: High-fidelity discrete modeling of the HPA axis: a study of regulatory plasticity in biology
Source: BMC Syst Biol. 2018 Jul 17;12:76. doi: 10.1186/s12918-018-0599-1 (PMC6050677; doi:10.1186/s12918-018-0599-1)

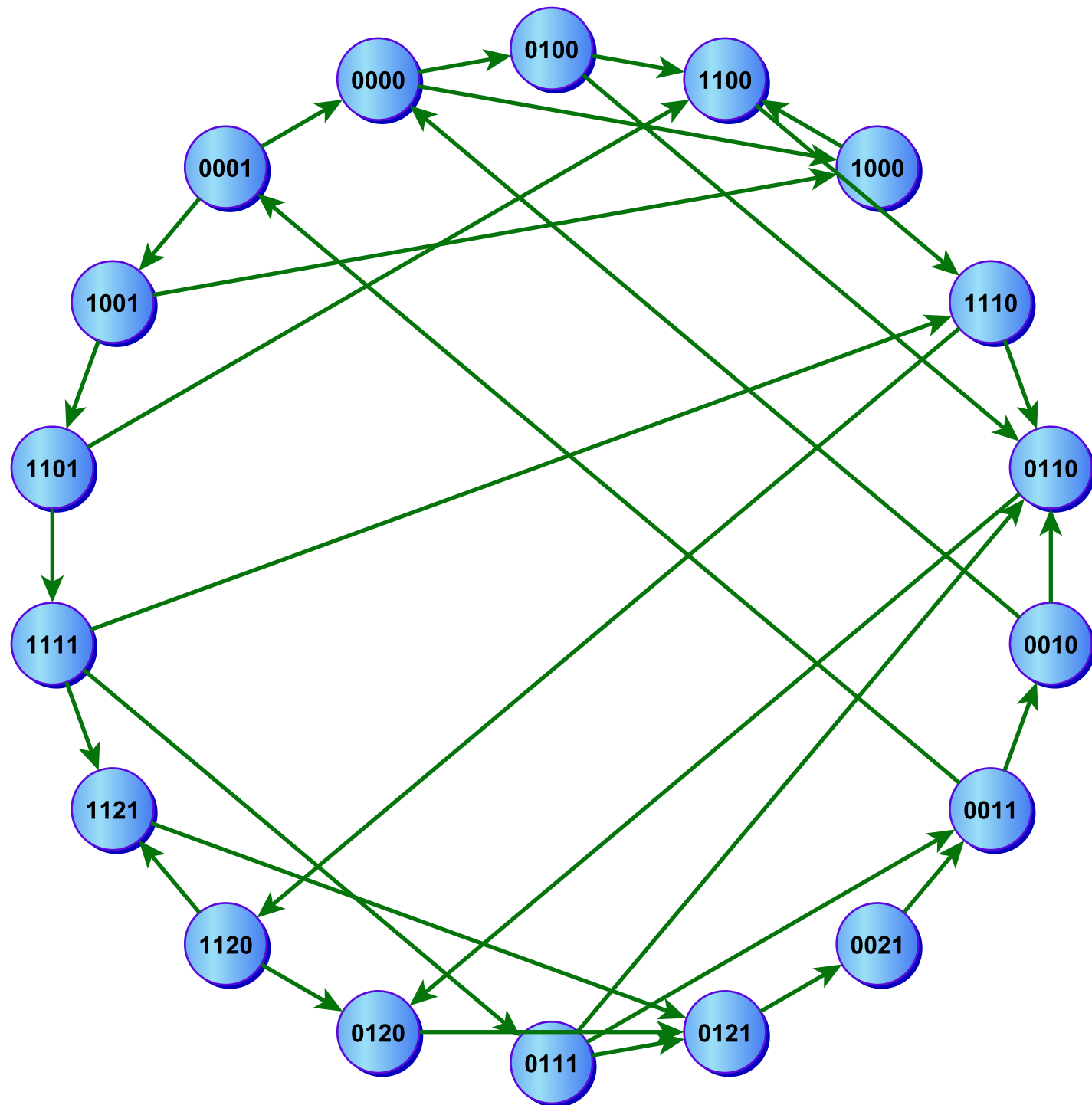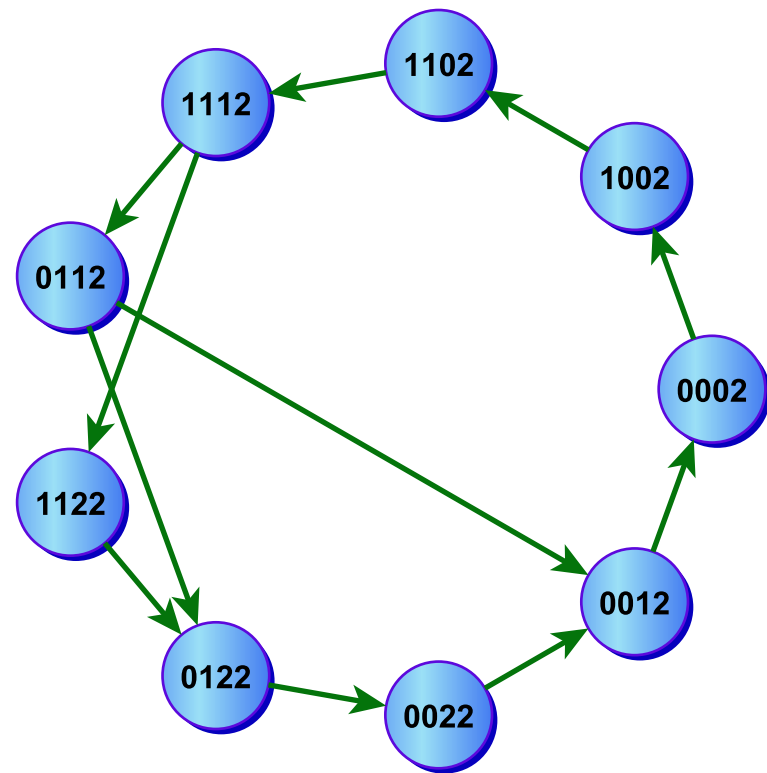

Supplement: Supplementary file 3 — Asynchronous Simulation. Asynchronous update of the network contains two complex cyclic attractors. Since under asynchronous update each state might have more than two successors several state nodes in the graph present with multiple out-bound edges. (PDF 52 kb) [file 12918_2018_599_MOESM3_ESM.pdf]

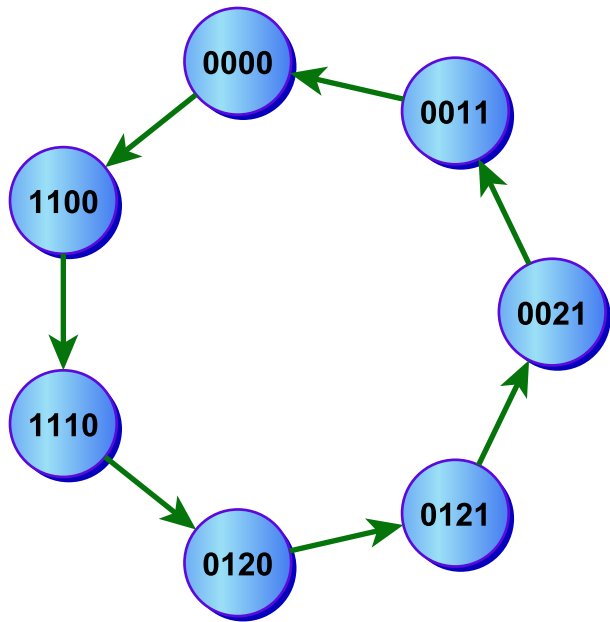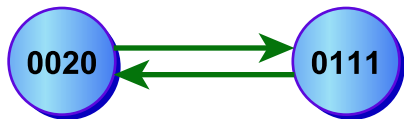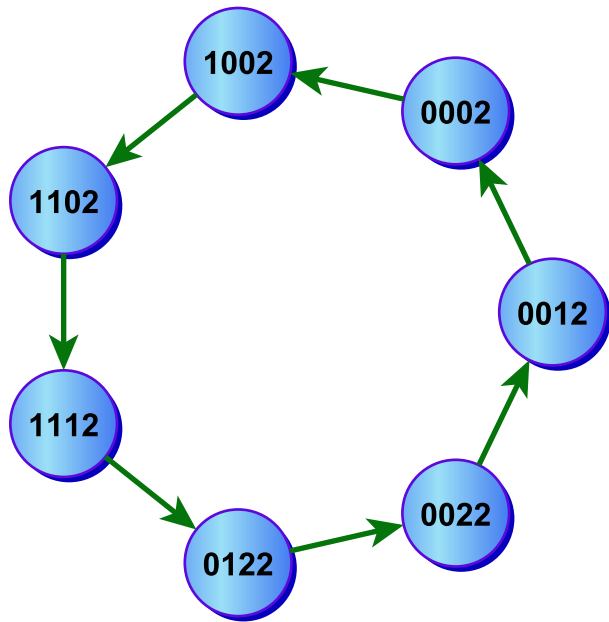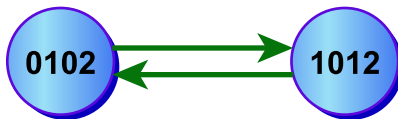

Supplement: Supplementary file 4 — Synchronous Simulation. Simulations with synchronous update where each node has only one successor state. It is well known that synchronous update can result in spurious cycles [12]. (PDF 36 kb) [file 12918_2018_599_MOESM4_ESM.pdf]

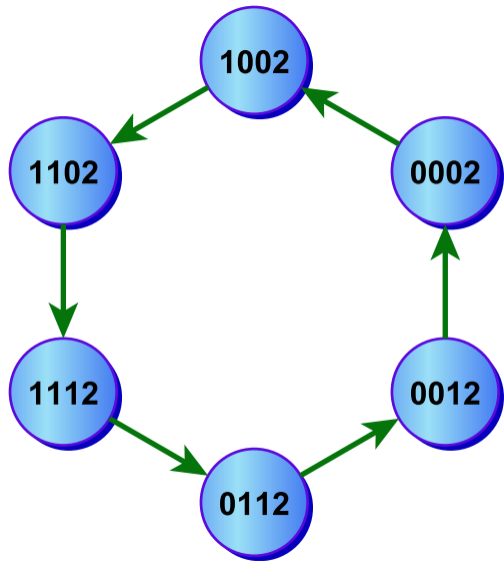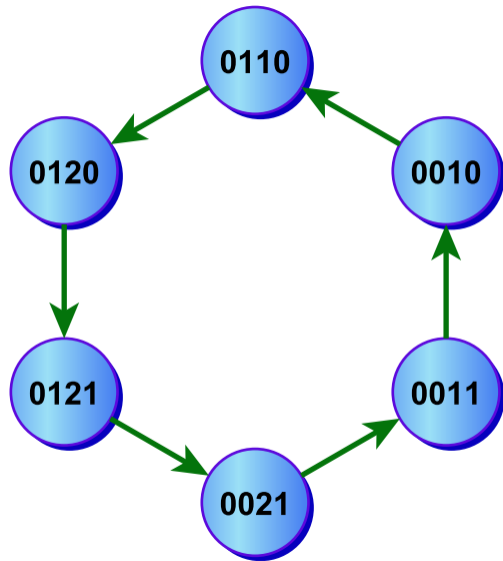

Supplement: Supplementary file 5 — Priority Memory Update. Simulations in priority class with memory. CORT is placed in a second priority class update and the rest of the parameters in the model have a higher priority and frequency of update. (PDF 24 kb) [file 12918_2018_599_MOESM5_ESM.pdf]
