# Supplementary material for: High-fidelity discrete modeling of the HPA axis: a study of regulatory plasticity in biology
Source: BMC Syst Biol. 2018 Jul 17;12:76. doi: 10.1186/s12918-018-0599-1 (PMC6050677; doi:10.1186/s12918-018-0599-1)
Supplement: Supplementary file 6 — Discrete Mathematical Equations Governing HPA axis. Details the derivation of discrete equations employed for the simulation of HPA axis. (PDF 132 kb) [file 12918_2018_599_MOESM6_ESM.pdf]

Additional File 1

Hooman Sedghamiz

December 6, 2017

Discrete Mathematical Equations Governing HPA axis

According to Chaouiya et al. [1], the image function might be written as;

$$y_i = \sum_{I \subseteq q(i)} K_{i.I} \left[\prod_{j \in I} S^{u_{ij}}(x_j, w_{ij}) \prod_{j \in q(i) \setminus I} \big(1 - S^{u_{ij}}(x_j, w_{ij})\big) \right]. \tag{1}$$

The image function simply states that when there is no active interaction modulating x_i, the image is equal to its basal value $K_{i\varnothing} = D(k_{i\varnothing})$ and when more than one interaction is applied concurrently, the image is equal to their joint logical parameter (e.g. $K_{1.12} = D(k_{11} + k_{12})$ when both variable 1 and 2 are modulating variable 1). According to Eq. 1, the corresponding ODE model of the HPA axis can be expressed in a generalized discrete form as follows,

$$\begin{aligned} y_2 = & \Big[K_{2\varnothing}\big(1 - S^{\alpha_{21}}(x_1, \theta_{21})\big)\big(1 - S^{\alpha_{24}}(x_4, \theta_{24})\big) \Big] \\ & + \Big[K_{21}\big(S^{\alpha_{21}}(x_1, \theta_{21})\big)\big(1 - S^{\alpha_{24}}(x_4, \theta_{24})\big) \Big] \\ & + \Big[K_{24}\big(1 - S^{\alpha_{21}}(x_1, \theta_{21})\big)\big(S^{\alpha_{24}}(x_4, \theta_{24})\big) \Big] \\ & + \Big[K_{2.14}\big(S^{\alpha_{21}}(x_1, \theta_{21})\big)\big(S^{\alpha_{24}}(x_4, \theta_{24})\big) \Big] \end{aligned} \tag{2}$$

$$\begin{aligned} y_3 = & \Big[K_{30}\big(1 - S^{\alpha_{32}}(x_2, \theta_{32})\big)\big(1 - S^{\alpha_{35}}(x_5, \theta_{35})\big) \Big] \\ & + \Big[K_{32}\big(S^{\alpha_{32}}(x_2, \theta_{32})\big)\big(1 - S^{\alpha_{35}}(x_5, \theta_{35})\big) \Big] \\ & + \Big[K_{35}\big(1 - S^{\alpha_{32}}(x_2, \theta_{32})\big)\big(S^{\alpha_{35}}(x_5, \theta_{35})\big) \Big] \\ & + \Big[K_{3.25}\big(S^{\alpha_{32}}(x_2, \theta_{32})\big)\big(S^{\alpha_{35}}(x_5, \theta_{35})\big) \Big] \end{aligned} \tag{3}$$

$$y_4 = \left[K_{40}\left(1 - S^{\alpha_{43}}(x_3, \theta_{43})\right)\right] + \left[K_{43}\left(S^{\alpha_{43}}(x_3, \theta_{43})\right)\right] \tag{4}$$

$$\begin{aligned} y_5 = &\left[K_{50}\left(1 - S^{\alpha_{54}}(x_4, \theta_{54})\right)\left(1 - S^{\alpha_{55}}(x_5, \theta_{55})\right)\right] \\ &+ \left[K_{55}\left(S^{\alpha_{55}}(x_5, \theta_{55})\right)\left(1 - S^{\alpha_{54}}(x_4, \theta_{54})\right)\right] \\ &+ \left[K_{54}\left(1 - S^{\alpha_{55}}(X_5, \theta_{55})\right)\left(S^{\alpha_{54}}(x_4, \theta_{54})\right)\right] \\ &+ \left[K_{5.54}\left(S^{\alpha_{55}}(X_5, \theta_{55})\right)\left(S^{\alpha_{54}}(x_4, \theta_{54})\right)\right] \end{aligned} \tag{5}$$

Where the indices 1 - 5 correspond to the state variables Stress, CRH, ACTH, CORT and the receptor R respectively.

References

[1] C. Chaouiya, E. Remy, B. Moss, and D. Thieffry. Qualitative Analysis of Regulatory Graphs : A Computational Tool Based on a Discrete Formal Framework. *Positive Systems*, pages 119–126, 2003.
