# Supplementary material for: High-fidelity discrete modeling of the HPA axis: a study of regulatory plasticity in biology
Source: BMC Syst Biol. 2018 Jul 17;12:76. doi: 10.1186/s12918-018-0599-1 (PMC6050677; doi:10.1186/s12918-018-0599-1)
Supplement: Supplementary file 7 — Algorithms for Computation of Attractors. Pseudo-code and algorithm for computation of attractors. (PDF 151 kb) [file 12918_2018_599_MOESM7_ESM.pdf]

Additional File 3

Hooman Sedghamiz

December 6, 2017

Algorithms for detection of Attractors

We use a variant of Tarjans [1] algorithm in order to detect regular and cyclic attractors. Algorithm 1 represents the pseudo-code of the method. In this approach, instead of storing the whole universe of possible states we use lexicographical indexing. Functions ***LexToState()*** (line 9) and ***LexToInd()*** (line 6) convert a scalar lexicographical index to its corresponding vector of states and vice versa. Algorithm 1 employs function ***strongconnect()*** (detailed in Algorithm 2) in order to compute the attractors.

Algorithm 1: Computing Attractors

```
input  : ADJ, Regulatory graph adjacency list
         k, Logical parameters struct
         N, Max transcription level vector
         Delay, select (Asynch, Synch, Priority)
output: ATR, attractors
1  all_attractor(ADJ, k, N, Delay)
2  begin
       // initialize a struct V with the size of product(N[1 : end])
3      L ← product(N[1 : end]), S ← ∅, index ← 0, root_flag ← true
4      for i ← 1 : L do
5          ((V.onStack[i], V.outedge[i]) ← false), (V.index[i], V.lowlink[i] ← 0)
6      end
       // visit all vertices
7      for i ← 1 : L do
8          if V.index[i] = 0 then
               // Convert Lexicographical index to state vector
9              m ← LexToState(i, N)
10             ATR ← strongconnect(m)
11         end
12     end
13 end
```

Algorithm 2 details the modified Strongly Connected Component (SCC) method of Tarjan in order to identify the leafs of SCC which in turn are the attractors. It employs the

image() line 4 function that implements the image function (see Eq. 11 in the article) in order to compute the successors of a state (vertex) based on the selected delay scheme (synch, asynch and priority with memory). More details about these functions as well as a modified depth first search function which enumerates the STG is available in the appendix of this document.

Algorithm 2: Modified SCC algorithm

```
   input  : m, a node from graph
1  strongconnect(m)
2  begin
3      ((V.index[i], V.lowlink[i]) ← index), index ← index + 1
4      S.push(v), ATR ← ∅, V.onStack[i] ← true, w ← image(ADJ, k, m, Delay)
5      for i = 1 : w.size() do
6          j ← LexToInd(w[i], N)
7          if V.index[j] = 0 then
8              strongconnect(w[i])
9              V.lowlink[i] ← min(V.lowlink[i], V.lowlink[j])
10         else if V.onStack[j] = true then
11             V.lowlink[i] ← min(V.lowlink[i], V.index[j])
12         else
13             V.outedge[i] ← true
14         end
15     end
16     if (V.lowlink[i] = V.index[i]) then
17         initialize a new SCC
18         while w ≠ i do
19             w ← S.pop(), V.onStack[w] ← false
20             add w to SCC
21         end
22         if root_flag = true then
23             flag_attractor ← true
24             for i ← 1 : SCC.size() do
25                 if V.outedge[SCC[i]] = true then
26                     flag_attractor ← false
27                     break
28                 end
29             end
30         end
31         if flag_attractor then
32             Report ATR ← SCC as attractor
33         else
34             ATR ← ∅
35         end
36         root_flag ← true
37     end
38 end
```

References

[1] R. Tarjan. Enumeration of the Elementary Circuits of a Directed Graph, 1973.
